# High expression of miR-105-1 positively correlates with clinical prognosis of hepatocellular carcinoma by targeting oncogene NCOA1

**DOI:** 10.18632/oncotarget.14435

**Published:** 2017-01-02

**Authors:** Yu-Shui Ma, Ting-Miao Wu, Zhong-Wei Lv, Gai-Xia Lu, Xian-Ling Cong, Ru-Ting Xie, Hui-Qiong Yang, Zheng-Yan Chang, Ran Sun, Li Chai, Ming-Xiang Cai, Xiao-Jun Zhong, Jian Zhu, Da Fu

**Affiliations:** ^1^ Department of Nuclear Medicine, Shanghai Tenth People's Hospital, Tongji University School of Medicine, Shanghai 200072, China; ^2^ Shanghai Engineering Research Center of Molecular Therapeutics and New Drug Development, College of Chemistry and Molecular Engineering, East China Normal University, Shanghai 200062, China; ^3^ Department of Radiology, the Fourth Affiliated Hospital, Medical University of Anhui, Hefei 230601, China; ^4^ Tissue Bank, China-Japan Union Hospital, Jilin University, Changchun 130033, China; ^5^ Department of Pathology, Shanghai Tenth People's Hospital, Tongji University School of Medicine, Shanghai 200072, China; ^6^ Department of Medical Oncology, the First Affiliated Hospital of Nanchang University, Nanchang 330006, China; ^7^ Department of Digestive Surgery, Rui Jin Hospital, Shanghai Jiao Tong University School of Medicine, Shanghai 200025, China; ^8^ Central Laboratory for Medical Research, Shanghai Tenth People's Hospital, Tongji University School of Medicine, Shanghai 200072, China

**Keywords:** miR-105-1, HCC, biomarker, survival, target

## Abstract

Increasing evidence supports that microRNA (miRNA) plays a significant functional role in cancer progression by directly regulating respective targets. In this study, the expression levels of miR-105-1 and its target gene were analyzed using genes microarray and hierarchical clustering analysis followed by validation with quantitative RT-PCR in hepatocellular carcinoma (HCC) and normal liver tissues. We examined the expression of nuclear receptor coactivator 1 (NCOA1), the potential target gene of miR-105-1, following the transfection of miR-105-1 mimics or inhibitors. Our results showed that miR-105-1 was downregulated in HCC tissues when compared with normal liver tissues and patients with lower miR-105-1 expression had shorter overall survival (OS) and progression free survival (PFS). Moreover, NCOA1 was confirmed to be a direct target of miR-105-1. Furthermore, concomitant high expression of NCOA1 and low expression of miR-105-1 correlated with a shorter median OS and PFS in HCC patients. In conclusion, our results provide the first evidence that NCOA1 is a direct target of miR-105-1 suggesting that NCOA1 and miR-105-1 may have potential prognostic value and may be useful as tumor biomarkers for the diagnosis of HCC patients.

## INTRODUCTION

Hepatocellular carcinoma (HCC) is one of the world's most common malignant tumors and is characterized by poor prognosis and high mortality rates [1]. More than 80% of the 782,000 estimated new HCC cases in 2012 occurred in less developed regions of the world, and its incidence continues to increase worldwide, including most developed countries [2]. While recent advancements in medical imaging have resulted in early diagnosis of HCC patients, clinical prognosis of HCC remains dire despite the use of targeted therapies [3–5]. Hence, there is a need to deepen our understanding of the molecular basis of HCC, including disease occurrence and development.

MicroRNAs (miRNAs), a class of non-coding RNA molecules of 21-24 nucleotides that regulate the expression of target genes in a post-transcriptional manner, can effectively modulate various biological processes, including cell proliferation, migration, differentiation, and apoptosis [6–8]. Currently, miRNA profiling has greatly improved with assays that have become less cumbersome, much faster, and cheaper. Furthermore, the stability of miRNA in blood and other body fluids renders their detection well suited for early diagnosis and outcome prediction of cancer patients, as well as their potential as therapeutic targets [5, 9]. Previous studies have identified various miRNAs in HCC patients, including miR-1, miR-9, miR-15b, miR-130b, miR-16, miR-18a, and miR-21 [10–13]. Although miR-105-1 has been detected in many tumors, such as breast cancer, prostate tumor, human glioma, and gastric cancer [14–17], its role and clinical significance have not been clarified in HCC. Therefore, in this study, we explored the clinical significance and target gene of miR-105-1 as a prognostic biomarker for HCC patients.

## RESULTS

### MiRNAs microarray analysis using GEO database

**Figure 1:**
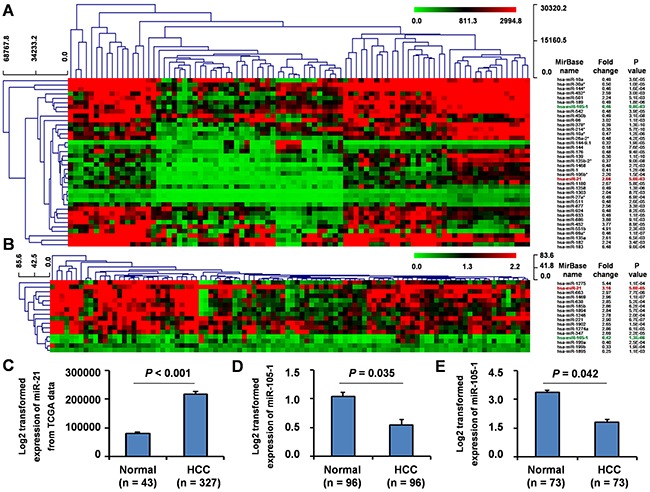
Differential expression of miR-105-1 in HCC using the GEO datasets Clustered analysis of differential expression of miRNAs (fold change ≥ 2 or ≤ 0.5, P < 0.01) in GSE36915 from GEO database including 68 HCC and 21 non-tumor liver tissues **A**. and GSE62044-13481 from GEO database including 100 fresh tissues of HCC patients and paired non-tumor liver tissues **B**. miR-105-1 expression in 327 HCC and 43 non-tumor liver tissues from TCGA data **C**. GSE22058-GPL10457 from GEO database including 96 pairs of HCC and non-tumor liver tissues **D**. and GSE21362 from GEO database including 73 pairs of HCC and non-tumor liver tissues **E**. Normal, healthy controls; HCC, hepatocellular carcinoma. Data shown as mean values with 95% confident intervals. P values were calculated by Mann-Whitney-Wilcoxon test.

To investigate the differential miRNA expression between HCC tissues and non-tumor liver tissues, we downloaded one group of data from GEO database (GSE36915) which included 68 HCC and 21 non-tumor liver tissues. We found 14 upregulated and 24 downregulated miRNAs (fold change (FC) ≥ 2 or ≤ 0.5, *P* < 0.01) (Figure 1A) in HCC tissues when compared with normal liver tissues. Among the downregulated miRNAs, miR-105-1 had an FC score of 0.46 (*P* = 9.8E-03).

Furthermore, we downloaded and analyzed another group of miRNAs microarray dataset (GSE62044-13481) from GEO database from 100 fresh tissues of HCC patients and paired non-tumor liver tissues. The result showed that a total of 16 miRNAs displayed at least a 2-fold increased or 0.5-fold decreased in expression levels at the *P* < 0.01 level (Figure 1B), of which 12 miRNAs were upregulated and 4 were downregulated. In this dataset, miR-105-1 was again identified with an FC score of 0.42 (*P* = 1.3E-06).

In all, comparing the miRNAs from both GEO datasets, we consistently identified miR-21 was upregulated, while miR-105-1 was downregulated (Figure 1A–1B).

### TCGA database analysis to validate miRNA-105-1 expression in HCC

To validate these findings, we downloaded miRNAs expression data of 327 HCC and 43 non-tumor liver tissues from TCGA. The results demonstrated that the levels of miRNA-21 displayed a 2.69-fold increase (*P* < 0.05) (Figure 1C). However, the expression, clinical significance and molecular mechanisms of miR-21 in HCC had been widely reported, therefore we did not explore the expression and clinical significance of miR-21 in HCC further in the current study.

However, the analysis of TCGA database indicated that there was no significant difference of miR-105-1 expression level between HCC and non-tumor liver tissues. We posit that this may be a new differential expression miRNA identified from GEO database.

Therefore, to further validate our results, we downloaded and analyzed two additional groups of datasets (GSE22058-GPL10457 and GSE21362) from GEO database, which included 96 and 73 pairs HCC and non-tumor liver tissues, respectively. These results also demonstrated that the levels of miR-105-1 was downregulated (FC = 0.52 and 0.61, *P* = 0.035 and 0.042, respectively) (Figure 1D, E) in HCC tissues when compared with those in non-tumor liver tissues.

### Validation of downregulated miR-105-1 levels using qRT-PCR

**Figure 2:**
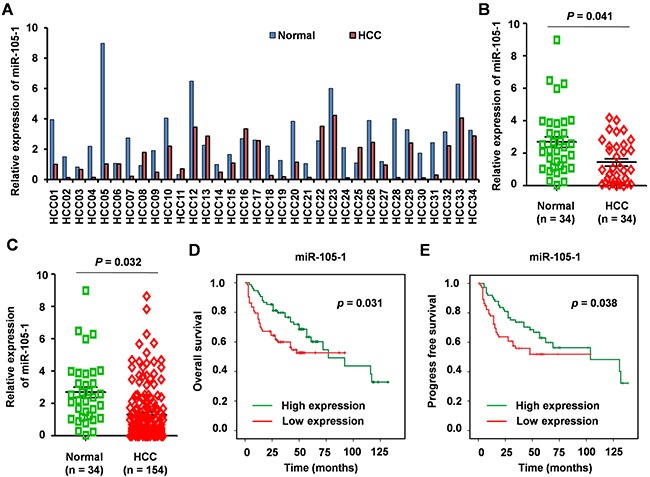
The expression levels and clinical significance of miR-105-1 in HCC **A**. qRT-PCR to quantify miR-105-1 level in 34 paired HCC and adjacently normal tissues. **B**. miR-105-1 expression levels in HCC vs. adjacent non-tumor tissue (n = 34). **C**. miR-105-1 levels between 154 HCC tissues and 34 adjacently normal liver tissues. Dot plots illustrate median values with 25th and 75th percentiles with whiskers to 10th and 90th percentiles. P values were calculated by Mann-Whitney-Wilcoxon test. Kaplan-Meier survival analysis to evaluate the prognostic value of miR-105-1 expression for OS **D**. and PFS **E**. of HCC patients.

Next, we validated the expression levels of miR-105-1 in HCC tissue samples using RT-PCR in 154 HCC biopsies, 34 of which were pairs of tissue from para-carcinoma tissues. MiR-105-1 level was downregulated in most of the 34 paired tissues (FC = 0.59, *P* = 0.041) (Figure 2A, 2B). When compared with normal liver tissues, the levels of miR-105-1 displayed a 0.51-fold decrease (*P* = 0.032) in 154 HCC biopsies (Figure 2C).

### The relationship between miR-105-1 and prognosis in HCC patients

We used Kaplan-Meier survival analysis to evaluate the prognostic value of miR-105-1 expression in HCC for OS and PFS. The results showed that lower expression of miR-105-1 was associated with shorter OS (*P* = 0.031) and PFS (*P* = 0.038) (Figure 2D).

### Gene microarray analysis of HCC patients

**Figure 3:**
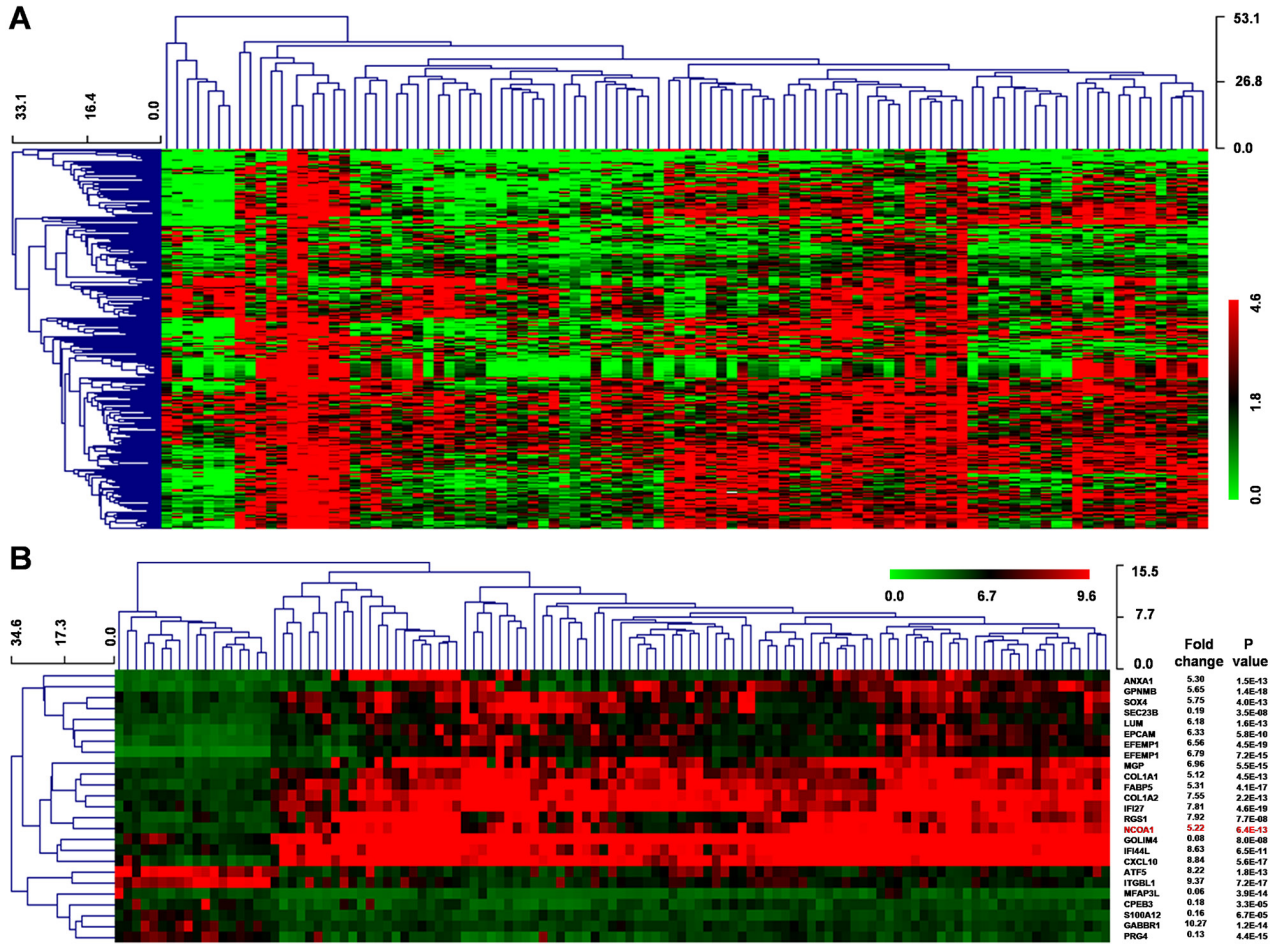
Differential gene expression of HCC using the GEO datasets **A**. Clustered analysis of 286 dysregulated genes (FC ≥ 5 or ≤ 0.2, P < 0.001) from GSE62044-GPL6480 data including 100 paired HCC and normal liver samples. **B**. Clustered analysis of differential gene expression from GSE14323 data, which included 19 normal liver tissues, 41 liver cirrhosis samples, 17 cirrhosis with HCC tissues, and 38 HCC samples. P values were calculated by Mann-Whitney-Wilcoxon test.

Next, we downloaded gene expression microarray data from GEO datasets (GSE62044-GPL6480) from 100 fresh tissues of HCC patients and paired non-tumor liver tissues. Our results indicated that the differential expression of 286 genes was extremely significant (FC ≥ 5 or ≤ 0.2, *P* < 0.001) (Figure 3A).

Moreover, we downloaded another mRNA expression microarray data (GSE14323) that included 19 normal liver tissues, 41 liver cirrhosis samples, 17 cirrhosis with HCC tissues, and 38 HCC samples. Herein, we identified 651 genes had extreme significant differential expression (FC ≥ 5 or ≤ 0.2, *P* < 0.001) and 25 genes had a similar change pattern in normal, cirrhosis, cirrhosis-HCC and HCC group (Figure 3B). Moreover, we found 49 common genes in these two differential expression genes.

### Prediction of target gene of miR-105-1

To investigate the genes targeted by miR-105-1, we used three target genes prediction websites (Targetscan, MIRDB, and PicTar) to forecast target genes of miR-105-1. The result showed that there were 35 common predicted target genes.

Our *in silico* analysis and qRT-PCR validation showed that miR-105-1 expression levels were significantly downregulated in HCC liver biopsies compared to normal tissues. Additionally, decreased miR-105-1 expression was associated with HCC progression and poor prognosis in patients, which suggested that miR-105-1 had a potential antitumor function. Therefore, we downloaded and screened candidates from Candidate Cancer Gene Database (http://ccgd-starrlab.oit.umn.edu/about.php) to identify an oncogene as the potential target of miR-105-1.

**Figure 4:**
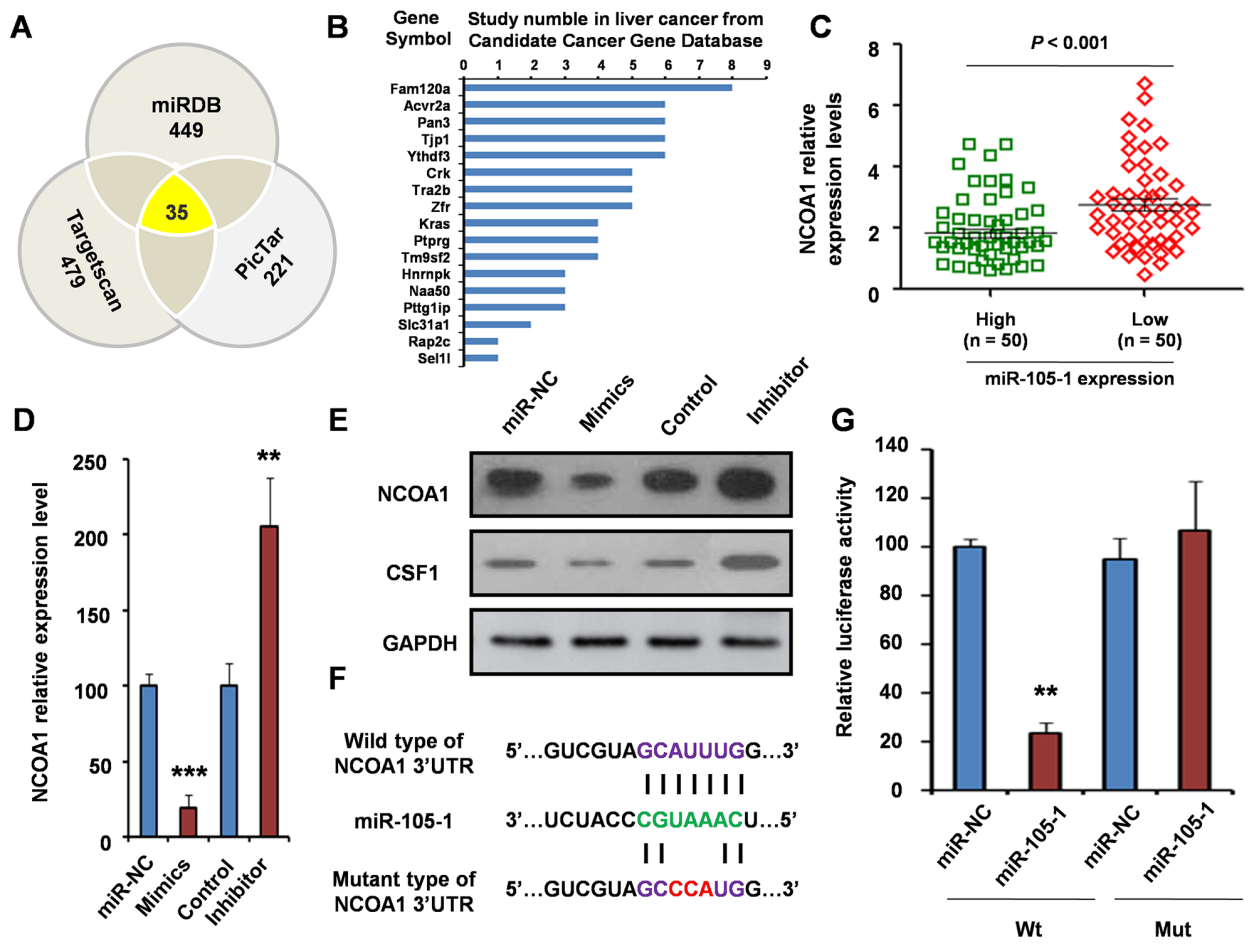
Validation of NCOA1 as a direct target of miR-105-1 **A**. miR-105-1 target prediction using three target genes prediction programs (Targetscan, miRDB, and PicTar). **B**. Among 35 common predicted target genes, 17 genes were reported to be involved in liver cancer. **C**. The relationship of NCOA1 levels with the expression of miR-105-1. Dot plots illustrate median values with 25th and 75th percentiles with whiskers to 10th and 90th percentiles. P values were calculated by Mann-Whitney-Wilcoxon test. qRT-PCR **D**. and Western blotting **E**. were used to measure the mRNA and protein level of NCOA1 after treatment of miR-105-1 mimics (overexpression) or inhibitor (knockdown). **F**. A schematic diagram of the miR-105-1 of 3 ’UTR in NCOA1 binding site and mutation site. **G**. Luciferase activity assay of pGL3-NCOA1-3’UTR reporter co-transfected with miR-105-1 mimic or miR-105-1-mut oligonucleotides in HeLa cells. Data shown are the means ± SD of three independent experiments. Statistical analyses were performed with one-way ANOVA (** = P < 0.01; *** = P < 0.001).

Our results showed that 21 genes in the CCGD database were among 35 common prediction target genes. However, of these 21 genes, 17 had has been reported associated with liver cancer (Figure 4B). The remaining four target genes (*ITGA6*, *NCOA1*, *Pou4f1* and *ST8SIA3*) had not been previously reported in HCC and only *NCOA1* was included in 49 common genes in the two differential expression genes thus piqued our interest (Figure 3A, 3B and 4A). Therefore, we sought to further analyze the correlation between NCOA1 and the expression of miR-105-1 in HCC patients.

Our results showed that NCOA1 expression was negatively correlated with miR-105-1 expression. The expression level of NCOA1 was downregulated in the miR-105-1-high expression group, and upregulated in the miR-105-1-low expression group (FC = 1.52, *P* < 0.001) (Figure 4C). Therefore, we hypothesized that NCOA1 was likely a target of miR-105-1.

### Identification of NCOA1 as a target of miR-105-1

To further confirm the relationship between NCOA1 and miR-105-1, we detected the NCOA1 mRNA and protein levels after treatment of miR-105-1 mimics or inhibitor in HeLa cells.

Compared to control group, the expression level of NCOA1 was lower in miR-105-1 mimics group (*P* < 0.001) and was higher in the miR-105-1 inhibitor group (*P* < 0.01) (Figure 4D), which suggested that NCOA1 were regulated by and negatively correlated with miR-105-1. Then, to further test the regulation role of miR-105-1 on NCOA1 at the protein level, we used Western blotting to measure the levels of NCOA1 protein and a reported downstream protein AKT1 [18] after miR-105-1 overexpression or knockdown. The results suggested that the increase in miR-105-1 levels significantly decreased NCOA1 protein expression and had the same tendency in AKT1 and vice versa (Figure 4E).

Using bioinformatics analysis, we found that miR-105-1 contained specific binding sequence of the 3’-UTR region of *NCOA1* gene. The 3’-UTR binding site and mutation site of miR-105-1 of *NCOA1* gene are shown in Figure 4F. We performed a luciferase reporter assay to further verify whether miR-105-1 directly targeted *NCOA1*. As shown in Figure 4G, ectopic expression of miR-105-1 decreased the luciferase activity of the 3’-UTRs of *NCOA1*. However, miR-105-1 mutant containing three altered nucleotides in the seed sequence did not have an inhibitory effect on luciferase activity.

### Biological role of miR-105 and NCOA1 in HCC progression

**Figure 5:**
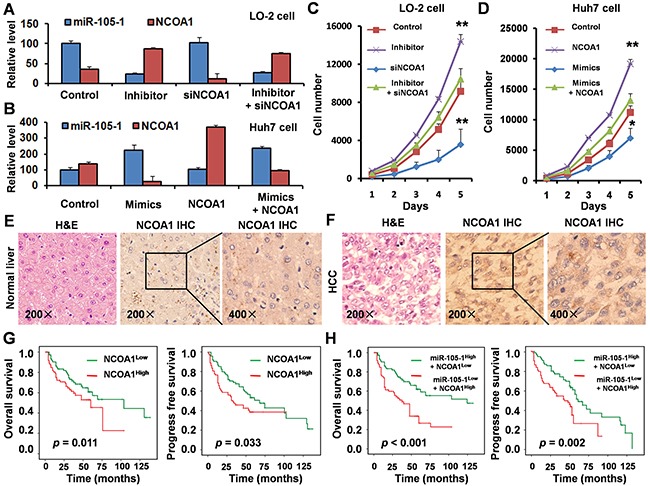
Biological role and clinical significance of miR-105 and NCOA1 in HCC **A**. qRT-PCR measurement of the levels of miR-105-1 and NCOA1 mRNA in LO-2 cells treated with negative control, miR-105-1 inhibitor and/or siNCOA1. Data shown are the means ± SD of three independent experiments. **B**. qRT-PCR measurement of the levels of miR-105-1 and NCOA1 mRNA in Huh7 cells treated with negative control, miR-105-1 mimics and/or NCOA1 expression vector. **C**. The LO-2 cell counts in 96-well plate after transfection with negative control, miR-105-1 inhibitor and/or siNCOA1 at the indicated day. **D**. Huh7 cell counts in 96-well plate after transfection with negative control, miR-105-1 mimics and/or NCOA1 expression vector at the indicated day. Data shown are the means ± SD of three independent experiments. Statistical analyses were performed with one-way ANOVA (* = P < 0.05; ** = P < 0.01). Immunohistochemical staining in paraffin sections of 154 paired normal liver **E**. and HCC tissues **F**. **G**. Kaplan-Meier survival analysis was used to evaluate the prognostic value of NCOA1 expression in HCC for OS and PFS. **H**. Kaplan-Meier survival analysis was used to evaluate the prognostic value of NCOA1 and miR-105-1 expression in HCC for OS and PFS.

To investigate the biological role of miR-105 in HCC progression, we performed loss-of-function studies using a miR-105-1 inhibitor and siNCOA1 on the normal liver cell lines LO-2 (Figure 5A). As shown in Figure 5C, suppression of miR-105-1 significantly enhanced the growth rate of LO-2 cells transfected with the miR-105-1 inhibitor compared with the negative control-transfected cells. However, cellular growth assay revealed that following siNCOA1 transfection, the growth rate of LO-2 cells was significantly inhibited when compared to the control group. There was no a dramatic change in cellular growth between among the LO-2 cells with miR-105-1 inhibitor and siNCOA1 transfection and negative control-transfected cells. Taken together, these results suggested that miR-105-1 downregulation promoted the proliferation of HCC cells and knockdown of NCOA1 inhibited HCC cell proliferation *in vitro*.

Next, we established the HCC cell line Huh7 to stably express miR-105-1, NCOA1, or vector (Figure 5B). The result of cellular growth assay showed that overexpression of miR-105-1 significantly inhibited the growth rate of Huh7 cells compared with control cells and overexpression of NCOA1 significantly promoted the growth rate of Huh7 cells (Figure 5D). There was no a dramatic change in cellular growth between Huh7 cells with miR-105-1- and NCOA1-overexpression and Huh7 cells transfected with vector control. These results suggested that miR-105-1 upregulation inhibited HCC cell proliferative capacity and overexpression of NCOA1 promoted HCC cell proliferation *in vitro*.

### Immunohistochemistry and clinical significance of NCOA1

To detect the expression levels of NCOA1 in HCC patients, immunohistochemical staining was performed in paraffin sections of 154 HCC tissues. The results showed that NCOA1 was highly expressed in HCC tissues when compared with normal liver tissues (Figure 5E, 5F).

Kaplan-Meier survival curves were plotted and log rank analysis was performed to evaluate the prognostic value of NCOA1 expression for patients with HCC. Our results indicated that NCOA1 expression was positively correlated with lower OS (*P* = 0.011) and PFS (*P* = 0.033) (Figure 5G) in HCC patients.

Since the data had suggested that NCOA1 expression level was negatively correlated with miR-105-1 expression, and NCOA1 was a direct target of miR-105-1, we further examined the prognostic value of NCOA1 expression together with miR-105-1 levels using multivariate analysis of OS and PFS by Kaplan-Meier survival analysis. The results showed that HCC patients with high NCOA1 expression and low miR-105-1 levels had significantly decreased OS (*P* < 0.001) and PFS (*P* = 0.002) (Figure 5H), which suggested that NCOA1 and miR-105-1 might have potential prognostic value and could be useful as tumor biomarkers for the diagnosis of HCC patients.

## DISCUSSION

HCC remains one of the most lethal forms of cancer in the world [19]. HCC patients have high malignancy and poor prognosis, especially in patients who fail first-line systemic therapy [20]. Thus, there is an urgent need to identify new diagnostic and prognostic makers for HCC patients. In previous studies, several pathogen-associated molecular and signaling pathways have been identified [21–24]. In addition, miRNAs, which can function as either tumor suppressors or oncogenes, are a sophisticated tool in therapeutics and diagnostics, and remain a prime focus among cancer researchers.

Several miRNAs have been identified to play a critical role in regulating HCC tumorigenesis and metastasis signaling networks [25–28]. Elucidating the underlying mechanisms of miRNA in liver carcinogenesis may improve diagnostic and therapeutic strategies for HCC. MiR-105-1 was previously identified as closely related to the circulation at the pre-metastatic stage and metastatic progression in early stage of breast cancer [16]. Besides, low expression of miR-105 may correlate with unfavorable clinical outcomes and be involved in tumorigenesis and aggressive progression of glioma [29]. Furthermore, miR-105 expression was found to be markedly downregulated in both HCC cell-lines and clinical HCC tissues and miR-105 could suppress the proliferation and tumorigenicity of HCC cells both *in vitro* and *in vivo* by deactivating the PI3K/AKT signaling pathway *via* targeting IRS1, AKT1 and PDK1, which suggests that miR-105 functions as a potential tumor suppressor [18].

However, the clinical significance of miR-105-1 in HCC and molecular mechanisms underlying the deactivation of its target genes still require elucidation. In this study, our *in silico* analysis and qRT-PCR validation showed that miR-105-1 expression levels were significantly downregulated in HCC liver biopsies compared to normal tissues. We also found that decreased miR-105-1 expression was associated with HCC progression and poor prognosis in patients, which is consistent with a previous study by Shen *et al*. [18].

To identify the target gene of miR-105-1 and potential molecular mechanism, we used three target genes prediction websites to forecast target genes and found that NCOA1was a direct target of miR-105-1.

NCOA1 has previously been reported to be overexpressed in breast cancer and its increased expression positively correlated with disease recurrence and metastasis through working with multiple transcription factors to, in turn, upregulate the expression of Twist1, ITGA5, CSF-1, SDF1 and CXCR4 [30]. Moreover, NCOA1 could promote angiogenesis in breast tumors by simultaneously enhancing both HIF1α- and AP-1-mediated VEGF-a transcription [31]. NCOA1, as the androgen receptor (AR) coactivator, was found to be involved in prostate cancer progression, while NCOA1 knockdown induced a significant decrease in migration and invasion through the upregulation of protein kinase D1 (PRKD1) [32]. Besides, NCOA1 expression was shown to have prognostic significance in advanced-stage head and neck carcinoma [33]. Nonetheless, the role and clinical significance of NCOA1 in HCC has not been investigated.

In the present study, we showed that NCOA1 expression was upregulated in HCC patients compared with normal liver tissue. Moreover, upregulated NCOA1 accompanied with downregulated miR-105-1 was associated with a shorter median OS.

Taken together, our findings indicate that miR-105-1 levels may play an essential role in HCC progression by targeting NCOA1 suggesting that NCOA1 and miR-105-1 have potential prognostic value as tumor biomarkers in HCC patients.

## MATERIALS AND METHODS

### Acquisition of clinical tissues samples

Fresh HCC samples were collected from patients undergoing surgical resection and classified according to the current WHO classification. Two experienced pathologists independently confirmed HCC diagnosis.

### Ethics statement

The study protocol including acquisition of tissue specimens was approved by the Ethical Committee of Shanghai Tenth People's Hospital (approval number SHSY-IEC-15-18). Each subject provided written informed consent before participating in this study.

### Bioinformatics analysis

The expression levels of miRNAs were investigated from GEO database (GSE36915, GSE62044-13481, GSE22058-GPL10457, GSE62044-GPL6480, GSE21362 and GSE14323) (www.ncbi.nlm.nih.gov/geo/). We downloaded miRNAs expression datasets from TCGA (http://cancergenome.nih.gov/) including 327 HCC and 43 paired non-tumor liver tissues. Hierarchical clustering was performed using the multiple experiment viewer (MeV) 4.7.1 software programs: (http://home.cc.umanitoba.ca/~psgendb/birchhomedir/doc/MeV/manual/StartingMEV.html). We used three target-gene prediction software Pictar (http://pictar.mdc-berlin.de/), Targetscan (http://www.targetscan.org), and miRDB (http://mirdb.org/miRDB/) to forecast several potential target genes of miR-105-1.

### Luciferase reporter assays

The human NOCA1 3’-UTR oligonucleotides containing the wild-type (Wt) or mutant (Mut) miR-105-1 binding site were sub-cloned into the *XhoI* and *NotI* sites of the pGL3 luciferase reporter plasmid vector (Promega, Madison, WI). For luciferase assay, HeLa cells were seeded in 24-well plates and cultured for 24 h; then, cells were co-transfected with either the Wt or Mut reporter plasmid. Forty-eight hours after transfection, luciferase assay was determined using the Dual-Luciferase kit (Promega, Madison, WI).

### RNA extraction

Total RNA was extracted from HCC and normal tissues using TRIzol regent (Invitrogen, Carlsbad, CA), according to the manufacturer's instructions. RNA acquisition was measured by spectrophotometer and the quality of all RNA samples was assessed by electrophoresis.

### Quantitative RT-PCR (qRT-PCR)

For qRT-PCR, cDNA was synthesized from total RNA (10ng), and quantitative PCR reactions were performed with the Taqman Universal PCR Kit (Life Technologies). GAPDH was used as the internal control. The 2^-ΔΔCT^ method was used to analyze the expression levels of miR-105-1.

### Western blotting

Total protein from HeLa cells on the condition of miR-105-1 mimics or inhibitor was extracted using cell lysis buffer to detect the expression levels of NCOA1 and a reported target molecule AKT1 [18]. Protein concentration was analyzed using standard procedures for Western blotting. After incubation with the appropriate horseradish peroxidase-conjugated secondary antibodies, the membranes were treated with an enhanced chemiluminescence reagent (Thermo Scientific, Dreieich, Germany), exposed to X-ray film (Kodak, Rochester, USA) and quantified by densitometry (Beckman, South Pasadena, Canada).

### Cell lines and transfection

Human HCC cell line Huh-7 and normal liver LO-2 cell lines were purchased from the Cell Bank of the Chinese Academy of Sciences (Shanghai, China) and cultured in DMEM media (Invitrogen, Carlsbad, USA) and supplemented with 10 % (v/v) fetal bovine serum, 100 U/ml penicillin, and 100 mg/ml streptomycin. Cell culture was conducted at 37 °C in a humidified 5% CO_2_ incubator. miR-105-1 mimics or anti-miR-105-1 oligonucleotide (Ambion, Austin, USA) was transfected using Lipofectamine 2000 reagent (Invitrogen, Carlsbad, USA) following the manufacturer's protocol. For NCOA1 over-expression, the human full lenght *NCOA1* cDNA was cloned into the pMSCV-hygro vector. The Huh-7 cells with stable over-expression of NCOA1 were polyclonal derivatives with hygromycin selection to avoid clonal variations in functional assays.

### Immunohistochemistry

Standard IHC and H&E staining were used to evaluate NCOA1 expression levels in 154 samples. Serial sections were stained in parallel with the primary antibody replaced by PBS as controls.

### Statistical analysis

Levels of miR-105-1 were summarized and recorded as mean ± standard deviation. The independent *t*- test was used to calculate the difference between two groups of data. The chi-square test was used to evaluate the difference among different groups. Kaplan-Meier curves and the log-rank test were used to analyze the overall survival (OS) or progression free survival (PFS) of HCC patients. Multivariate Cox proportional hazards regression models were performed to evaluate multiple characteristics in prognosis of HCC patients. All statistical analyses were performed using the SPSS 20.0 software program (SPSS Inc., Chicago, IL, USA).
